# Clinically suspected myocarditis in COVID‐19 patients: Case series and review of the literature

**DOI:** 10.1002/ccr3.5236

**Published:** 2021-12-26

**Authors:** Ahmad Amin, Seyed Parsa Eftekhar, Naghmeh Ziaie, Soudeh Roudbari, Pegah Salehi, Farzad Jalali, Iraj Jafaripour, Sanaz Ghaffari, Maryam Mohseni Salehi, Reza Ebadi

**Affiliations:** ^1^ Rajaie Cardiovascular Medical and Research Center Iran University of Medical Sciences Tehran Iran; ^2^ School of Medicine Babol University of Medical Sciences Babol Iran; ^3^ Department of Cardiology Babol University of Medical Sciences Babol Iran; ^4^ Department of Cardiology Shiraz University of Medical Sciences Shiraz Iran; ^5^ Department of Cardiology Ahvaz Jundishapur University of Medical Sciences Andimeshk Iran

**Keywords:** COVID‐19, echocardiography, myocarditis, NT‐proBNP, SARS‐CoV‐2

## Abstract

This Study describes eleven patients positive for severe acute respiratory syndrome coronavirus 2. In our cases, females and younger patients developed more severe disease. In contrast, improvement in left ventricular ejection fraction and N‐terminal prohormone brain natriuretic peptide within the first week of treatment contributed to promising outcomes.

## Graphical abstract

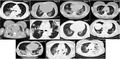

## 1 INTRODUCTION

Since the onset of the severe acute respiratory syndrome coronavirus 2 (SARS‐CoV‐2) pandemic, it was recognized that cardiac involvement might occur, confirmed by elevated cardiac troponin I. In addition, the presence of angiotensin‐converting enzyme 2 receptors (required for virus entry into cells) on cardiomyocytes could contribute to virus entry into cardiomyocytes. Moreover, mortality and morbidity were higher in patients with cardiovascular involvement.^1^, ^2^ Cardiac involvement includes various manifestations such as acute myocardial injury, arrhythmia, myocarditis, cardiogenic shock, and heart failure with varying severity.^3^ Nevertheless, knowledge about the characteristics of cardiac involvement and its mechanisms has not been evolved. Myocarditis is a rare complication in COVID‐19, contributing to a rapid deterioration of patients' condition.

Due to the low incidence and the fact that not all cases of myocarditis are recognized, the understanding of the mechanism and treatment is not thorough.^4^ The current study presents eleven COVID‐19 cases, clinically suspected of COVID 19–related myocarditis, their laboratory and echocardiographic findings, and their follow‐up over 3 months. Further prospective studies are required to identify the underlying mechanism, manifestations, and management of myocarditis in COVID‐19 patients.

## 2 CASE 1: LOSS OF CONSCIOUSNESS

**FIGURE 1:**
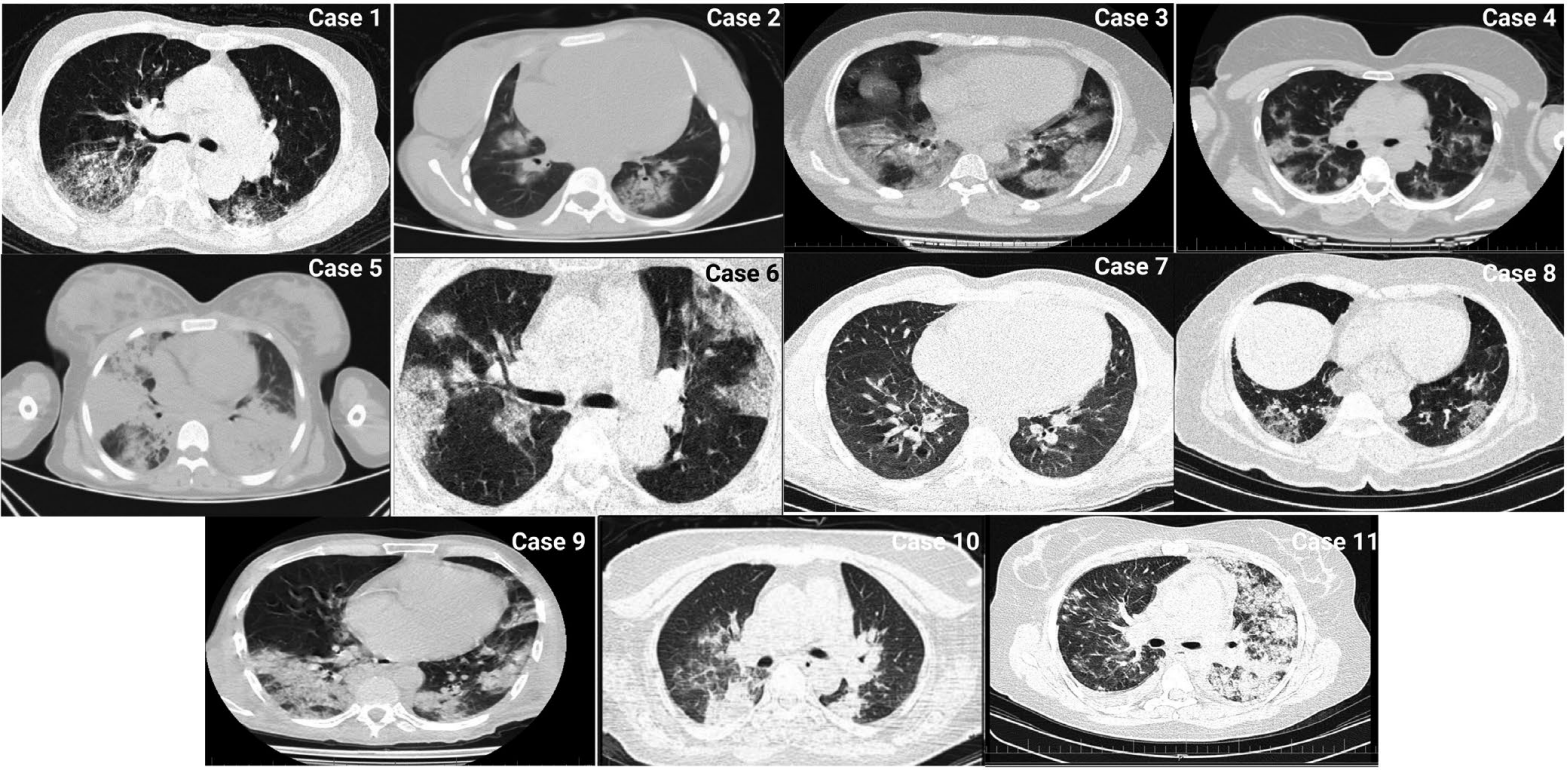
Chest CT scans show diffuse bilateral ground‐glass opacities (GGO) and basal consolidations in lung field

An 18‐year‐old woman (with no underlying disease) was admitted with a sudden loss of consciousness, fever, and headache that had occurred for 2 days before admission. At the time of admission, vital signs were as follows: body temperature = 36.8°C, systolic blood pressure = 80/60 mmHg, heart rate = 110/min, and O_2_ saturation = 80% without oxygenation. Glasgow Coma Scale (GCS) score was 11. She was intubated and treated with vasopressors. Also, rales were heard on pulmonary auscultation. Initial electrocardiogram (ECG) demonstrated sinus tachycardia (110/min) and low voltage QRS. Transthoracic echocardiography (TTE) indicated a small left ventricle (LV) with an end‐diastolic dimension of 42 mm, a left ventricular ejection fraction (LVEF) of 10% with global hypokinesis, a normal right ventricle (RV), and pulmonary arterial pressure (PAP), no pericardial effusion (PE) or thrombosis, and no significant valve lesions or dysfunction. Chest computed tomography (CT) scan revealed diffuse bilateral ground‐glass opacities (GGO) and basal consolidations (Figure 1). Moreover, the patient had a positive nasal and oropharyngeal polymerase chain reaction (PCR) test for COVID‐19. Laboratory findings are summarized in Table 1. The patient received intravenous immunoglobulin (IVIG; 1 g/kg), broad‐spectrum antibiotics (meropenem [500 mg] and doxycycline [200 mg/day]), high‐dose corticosteroid (methylprednisolone 1 g/day), and remdesivir for COVID‐19. However, she died due to cardiac arrest (less than 24 h after admission).

**TABLE 1:** Laboratory findings

	Case 1	Case 2	Case 3	Case 4	Case 5	Case 6	Case 7	Case 8	Case 9	Case 10	Case 11	Reference Range
White Blood Cells (Lymphocytes) (/µl)	18,000 (1800)	1600 (112)	16,400 (1148)	12,400 (1860)	1600 (128)	1300 (143)	10,700 (856)	11,000 (1650)	2400 (480)	1800 (252)	12,000 (2160)	4500–11,000
Hemoglobin (g/dl)	12.9	10.2	9.6	15	7.5	11	10.5	12.7	12	11	12	Male >13 Female >12
Platelets (10^4^/µl)	20.8	22.6	18.6	27.1	16.2	19	30.8	17.5	20.1	19.8	22	15,0000–45,0000
NT‐proBNP (pg/ml)	21,000	24,000	35,000	10,000	24,000	3000	21,000	4300	1200	22,000	23,000	<125
Blood Urea Nitrogen (mg/dl)	13	40	61	15	42	40	13	45	22	23	24	6–24
Creatinine (mg/dl)	1.1	1.6	2.1	0.9	0.9	1.2	2.5	1.3	1.1	1.1	0.8	0.74–1.35
Troponin (Times UNL)	Three	Two	Four	Two	Three	Four	Three	Two	Two	Three	Two	0–0.04 (ng/ml)
C–Reactive Protein (mg/L)	50	30	60	65	70	45	55	30	35	70	103	0.8–1
Procalcitonin (ng/ml)	2	4	2.5	0.2	5	0.2	2	0.8	0.2	2.2	<0.1	< .05
Interleukin−6 (pg/ml)	24	13	300	24	30	20	24	12	10	24	8	<6
D–Dimer (ng/ml)	1200	2400	7000	2400	4500	1500	3500	1400	1700	2400	1350	<250

Abbreviations: NT‐proBNP, N‐terminal‐pro hormone brain natriuretic peptide; UNL, upper normal limit.

## 3 CASE 2: ABDOMINAL PAIN

A 15‐year‐old girl (with no underlying disease) was admitted with mild dyspnea, abdominal pain, nausea, and fever that had occurred for 2 days before admission. At the time of admission, vital signs were as follows: body temperature = 39.8°C, systolic blood pressure = 90/50 mmHg, heart rate = 120/min, and O_2_ saturation = 95% without oxygenation. Cold limbs and thready pulse were obvious through physical examination. Moreover, the ECG revealed sinus tachycardia and inverted T wave in lateral limb and precordial leads. Simultaneously, an enlarged liver (146 mm) and elevated levels of liver enzymes (alanine aminotransferase [ALT] = 176 U/L, aspartate aminotransferase [AST] = 120 U/L, alkaline phosphatase [ALP] = 170 U/L, albumin = 4.5 g/dl, total bilirubin = 1 mg/dl, and direct bilirubin = 0.3 mg/dl) were observed that were suggestive of acute hepatitis. The TTE indicated severe ventricular (RV and LV) enlargement and dysfunction (LVEF = 5%–10%), moderate mitral regurgitation, PAP = 35 mmHg, and mild pericardial effusion. The patient had a positive nasal and oropharyngeal PCR test for COVID‐19, and the lung CT scan disclosed GGO as well as peripheral consolidations (Figure 1). The patient was treated with broad‐spectrum antibiotics (meropenem [500 mg q8 h for 5 days] and doxycycline [200 mg/day]), corticosteroids (methylprednisolone 1 mg/kg/day), and remdesivir. Ten days after admission, the patient was discharged from the hospital in a stable condition with normalization of white blood cell (WBC) count and liver enzymes, and the TTE exhibited no change compared to the first examination. However, an LVEF of 20% and a dysfunctional and enlarged RV were observed by follow‐up of the patient for 3 months. It is worth mentioning that the patient suffered from dyspnea during ordinary physical activities (functional class II).

## 4 CASE 3: DIFFUSE ST‐SEGMENT ELEVATION

**FIGURE 2:**
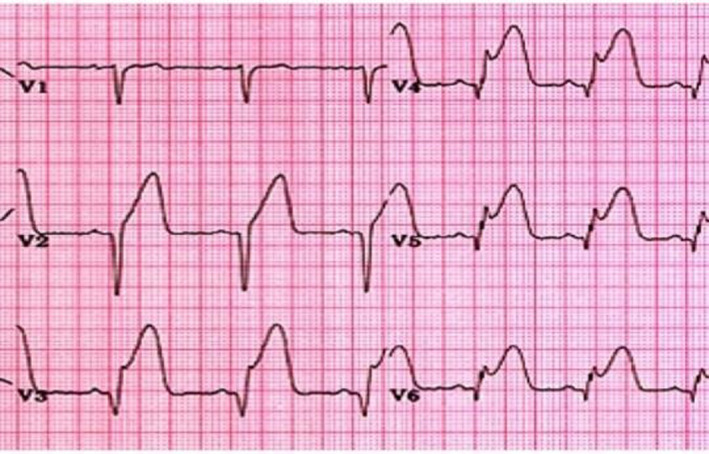
Case 3. ECG shows a normal sinus rhythm with diffuse anterior ST‐segment elevations in precordial leads

A 16‐year‐old boy was admitted with fever, diarrhea, and dry cough that had occurred for 1 week before admission. The first evaluation of the patient at the time of admission was as follows: low blood pressure (80/60 mmHg), non‐palpable pulse, and cyanosis, oxygen saturation was 85% without oxygenation, and the body temperature was 38°C. The ECG showed normal sinus rhythm with diffuse anterior ST‐segment elevations in precordial leads (Figure 2). Rapid improvement of the ECG abnormality resulted from carrying out vasopressors and intubation. Echocardiography represented left ventricular wall thickness, LVEF of 20%–25% with generalized hypokinesis, normal RV size (and function), and dilated inferior vena cava. Moreover, valvular structure and function were normal. The patient had a positive nasal and oropharyngeal PCR test for COVID‐19, and the lung CT scan disclosed GGO (Figure 1).

The patient received IVIG (1 g/kg), broad‐spectrum antibiotics (meropenem [500 mg] and doxycycline [200 mg/day]), high‐dose corticosteroid (methylprednisolone 1 g/day), and remdesivir for COVID‐19. Nonetheless, the patient died the next day after admission due to sudden bradycardia, hypotension, and cardiac arrest.

## 5 CASE 4: SYNCOPE

A 32‐year‐old man was admitted with syncope after an episode of chest pain accompanied by fever and myalgia that had occurred for 4 days before admission. At the time of admission, the GCS score equaled 13, and he was confused. Also, he complained about dyspnea and dry cough. Vital signs were as follows: body temperature = 36.8°C, systolic blood pressure = 85/60 mmHg, heart rate = 120/min, and O_2_ saturation = 80% without oxygenation. The ECG showed sinus tachycardia, low‐voltage limb leads, inverted T waves in two limb leads (L1 and aVL), and ST‐segment depression in three pericardial leads (V1–V3). The TTE revealed LVEF = 25%, an enlarged end‐diastolic diameter of the left ventricle, normal RV size (mild dysfunction), and normal valvular and pericardial function. Moreover, echocardiography demonstrated increased wall thickness. The patient had a positive nasal and oropharyngeal PCR test for COVID‐19 as well as the lung CT scan showed diffuse bilateral GGO suggestive of COVID‐19.

The patient received corticosteroids (methylprednisolone 1 mg/kg/day), broad‐spectrum antibiotics (meropenem [500 mg q8 h for 5 days] and doxycycline [200 mg/day]), and remdesivir for COVID‐19. The patient was discharged after 2 weeks with an ejection fraction of 45%. Follow‐up of the patient for 3 months revealed an LVEF of 45% and normal RV function.

## 6 CASE 5: ACUTE RESPIRATORY DISTRESS SYNDROME

A 14‐year‐old girl with a history of glucose‐6‐phosphate dehydrogenase (G6PD) deficiency was admitted with fever, cough, and dyspnea that had occurred since a week before admission. Moreover, she experienced loss of consciousness that had arisen about an hour before admission. Vital signs were as follows: body temperature = 38.2°C, systolic blood pressure = 60/50 mmHg, heart rate = 145/min, and O_2_ saturation = 60% without oxygenation. Also, the GCS score was 12. The ECG showed sinus tachycardia and low‐voltage limb leads. So, she was intubated immediately, and vasopressors were administered. The chest CT scan showed bilateral diffuse GGO and consolidations (Figure 1) as well as she had a positive nasal and oropharyngeal PCR test for COVID‐19. Furthermore, the patient was anemic (hemoglobin = 6 g/dl). Echocardiography demonstrated an LVEF of 15%, no pleural effusion, normal inferior vena cava, pulmonary arterial pressure of 30 mmHg, and normal LV and RV size. The patient received broad‐spectrum antibiotics (meropenem [500 mg] and doxycycline [200 mg/day]), high‐dose corticosteroid (methylprednisolone 1 g/day), and remdesivir for COVID‐19. However, the patient died in virtue of acute respiratory distress syndrome (ARDS).

## 7 CASE 6: VENTRICULAR ARRHYTHMIA

**FIGURE 3:**
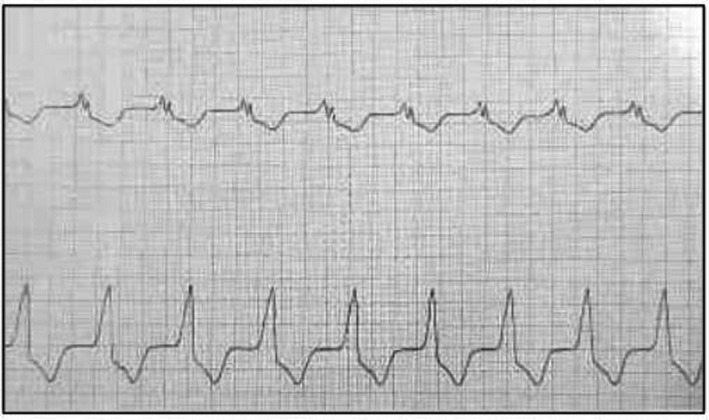
Case 6. ECG shows ventricular tachycardia

A 34‐year‐old woman was admitted with complaint of losing consciousness and she had also been suffering from fever and headache for six consecutive days. Vital signs were as follows: body temperature = 36.7°C, systolic blood pressure = 80/60 mmHg, heart rate = 114/min, and O_2_ saturation = 82% without oxygenation. Also, the GCS score equaled 8. The ECG monitoring revealed ventricular tachycardia (Figure 3); so, the direct current (DC) shock was administered and the patient was intubated. Echocardiography showed normal LV and RV size, moderate tricuspid and mitral regurgitation, systolic pulmonary artery pressure of 30 mmHg, and LVEF of 30%. The chest CT scan illustrated bilateral GGO and basal consolidations (Figure 1) as well as the patient had a positive nasal and oropharyngeal PCR test for COVID‐19. The patient received broad‐spectrum antibiotics (meropenem [500 mg q8 h for 5 days] and doxycycline [200 mg/day]), high‐dose corticosteroid (methylprednisolone 1 g/day), and remdesivir for COVID‐19. The patient was discharged after 3 weeks with LVEF of 40%–45% and normal cardiac biomarkers. Follow‐up of the patient for 3 months showed an LVEF of 45%–50%.

## 8 CASE 7: CARDIOGENIC SHOCK

A 22‐year‐old man (with no history of underlying disease) was admitted with dry cough and dyspnea that had developed for 5 days before admission. Vital signs were as follows: body temperature = 36.8°C, systolic blood pressure = 70/50 mmHg, heart rate = 120/min, and O_2_ saturation = 90% without oxygenation. The ECG showed sinus tachycardia. The patient had a positive nasal and oropharyngeal PCR test for COVID‐19, and pulmonary edema was observed by CT scan (Figure 1). Echocardiography showed LVEF of 30%–35%, left ventricular end‐diastolic diameter of 5 cm, moderate RV enlargement and dysfunction, dilated inferior vena cava, and mild left‐sided pleural effusion. The patient received broad‐spectrum antibiotics (meropenem [500 mg q8 h for 5 days] and doxycycline [200 mg/day]), high‐dose corticosteroid (methylprednisolone 1 g/day), and remdesivir for COVID‐19. The patient was discharged after 7 days with stable hemodynamics. Follow‐up of the patient for 3 months showed an LVEF of 45% and a normal cTnI value.

## 9 CASE 8: PERICARDIAL EFFUSION

**FIGURE 4:**
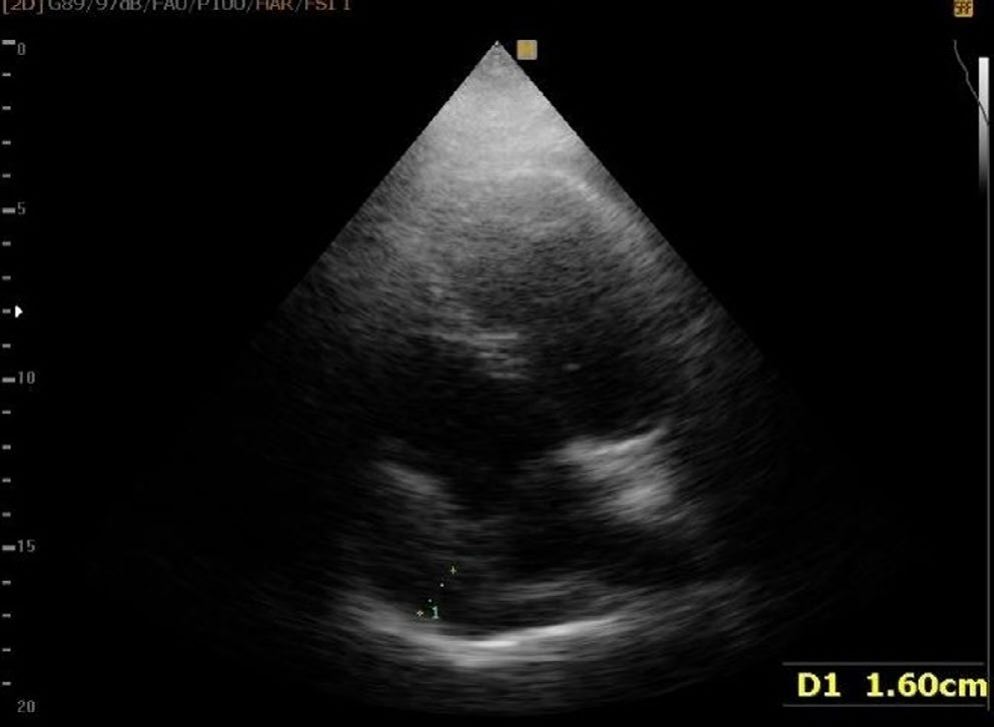
Case 8. Echocardiography shows normal LV size and function, without regional wall motion abnormalities, and a circumferential pericardial effusion without significant respiratory variation on mitral and tricuspid valves

A 45‐year‐old man was admitted with a fever that lasted for 2 weeks, dyspnea, and pleuritic chest pain continued for 1 week. Vital signs were as follows: body temperature = 38.5°C, systolic blood pressure = 100/70 mmHg, heart rate = 90/min and, O_2_ saturation = 85% without oxygenation (90% with nasal oxygen therapy). The ECG showed sinus tachycardia, no significant changes in the ST‐segment, low‐voltage waves in the limb leads, and QRS alternans. The TTE revealed LVEF of 45%, normal LV size and function without regional wall motion abnormalities, and circumferential pericardial effusion without significant respiratory changes in the mitral and tricuspid valves (Figure 4). Lung CT scan showed bilateral GGO suggestive of viral pneumonia (Figure 1) as well as the patient had a positive nasal and oropharyngeal PCR test for COVID‐19. The patient received broad‐spectrum antibiotics (meropenem [500 mg q8 h for 5 days] and doxycycline [200 mg/day]), corticosteroid (methylprednisolone 1 mg/kg/day), and remdesivir for COVID‐19. However, the pericardial effusion was resolved by the third day after admission. Oxygen saturation increased to 95% after 5 days (without supplemental oxygen) and he was discharged in a satisfying condition. Follow‐up of the patient for 3 months showed an LVEF of 45%, mild pericardial effusion, and normal right ventricular size and function.

## 10 CASE 9: CHEST PAIN

A 28‐year‐old man was admitted with chest pain, fever, and dry cough that had arisen for 3 days before admission. Vital signs were as follows: body temperature = 36.8°C, systolic blood pressure = 100/60 mmHg, heart rate = 92/min, and O_2_ saturation = 92% without oxygenation. The ECG illustrated sinus rhythm with ST‐segment elevation and Q waves in precordial leads. Also, angiography showed normal coronary arteries. Echocardiography revealed LVEF = 30%–35%, hypokinesis in the anterior wall, normal LV size as well as normal RV size and function. Lung CT scan showed a bilateral GGO pattern (Figure 1) as well as the patient had a positive nasal and oropharyngeal PCR test for COVID‐19. The patient received broad‐spectrum antibiotics (meropenem [500 mg q8 h for 5 days] and doxycycline [200 mg/day]), corticosteroid (methylprednisolone 1 mg/kg/day), and remdesivir for COVID‐19 for 7 days. It is worth mentioning that LVEF improved to 45%–50% after treatment. He recovered within 3 days and was discharged in a satisfying condition. Follow‐up of the patient for 3 months revealed LVEF of 45%–50% and a normal cardiac function.

## 11 CASE 10: TORSADE DE POINTES

**FIGURE 5:**
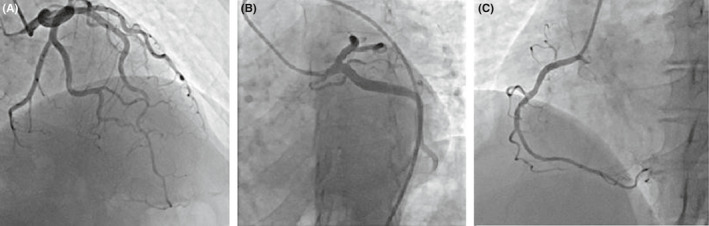
Case 10. Angiography shows a non‐significant (<50%) lesion in the middle part of the left anterior descending artery. (A) Left anterior descending artery, (B) Left circumflex artery, (C) Right coronary artery

A 51‐year‐old woman was admitted with cardiopulmonary arrest. The ECG showed torsade de pointes (Tdp). So, cardio‐version and lidocaine administration were carried out and she was successfully resuscitated after 20 min. Serum electrolytes were normal, and she received no QT‐prolonging agents. Lung CT scan exhibited bilateral GGO (Figure 1) as well as the patient had a positive nasal and oropharyngeal PCR test for COVID‐19. The TTE exhibited an LVEF of 35%, normal LV size, increased LV wall thickness (13 mm), no pericardial effusion, no valvular lesion, and no LV thrombus. The patient received IVIG (1 g/kg), broad‐spectrum antibiotics (meropenem [500 mg q8 h for 5 days] and doxycycline [200 mg/day]), high‐dose corticosteroid (methylprednisolone 1 g/day), and remdesivir for COVID‐19. So, her hemodynamic status improved after 12 h. Also, angiography displayed a non‐significant (<50%) lesion in the middle part of the left anterior descending artery (Figure 5). The TTE (after 10 days) revealed a 50% improvement of LVEF with a slight increase in LV wall thickness (11 mm) and the cTnI value had decreased. The patient was discharged in a satisfying condition after 10 days. Follow‐up of the patient over 3 months demonstrated an LVEF of 50%.

## 12 CASE 11: LEFT VENTRICLE THROMBUS

**FIGURE 6:**
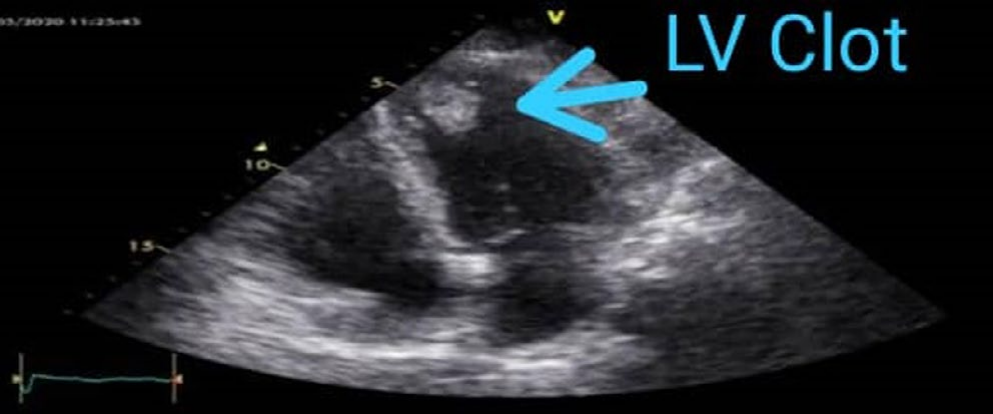
Case 11. Echocardiography shows a hypermobile clot in the LV (1.5 × 1.5 cm)

A 39‐year‐old woman was admitted with chest pain, orthopnea, and cough that had arisen for 1 week before admission. Also, she had a history of asthma. Vital signs were as follows: body temperature = 37.2°C, systolic blood pressure = 100/60 mmHg, heart rate = 125/min, and O_2_ saturation = 90% without oxygenation. The ECG showed sinus tachycardia. The patient had a positive nasal and oropharyngeal PCR test for COVID‐19 and the lung CT scan showed GGO in both lungs (Figure 1). Echocardiography represented an LVEF of 30%–35%, normal LV size, mild RV enlargement (restrictive diastolic pattern), moderate tricuspid and mitral regurgitation, and mild pericardial effusion. Cardiac magnetic resonance (CMR or cardiac MRI) imaging showed myocardial edema and hyperemia suggestive of active myocarditis. Also, coronary CT angiography was normal. Subepicardial and subendocardial fibrosis in the middle part of the inferoseptal wall was also present. Her vital signs and oxygen saturation (98% on room air) improved after dexamethasone administration for 3 days. Echocardiography revealed an LVEF of 45% and a hypermobile thrombus in the LV (1.5 × 1.5 cm; Figure 6). Although the cardiac surgeon suggested surgical removal of the thrombus, the patient refused the surgery. So, the patient was subjected to anticoagulation therapy (heparin 24,000 U/24 h). After 3 days, the thrombus had disappeared on the echocardiography, and there were no clues of embolism. The patient was discharged in a satisfying condition on β‐blocker, angiotensin‐converting enzyme (ACE) inhibitor, spironolactone, and warfarin. Follow‐up of the patient for 3 months showed an LVEF of 45%–50%.

## 13 DISCUSSION

In a post‐mortem histopathological study, the incidence of myocarditis was 7.2% (among 277 cases of COVID‐19).^5^ Although myocarditis does not seem to be very common in infected patients, its low prevalence may be due to the fact that cardiac MRI or biopsy was not performed in all patients. The current study presents eleven cases of clinically suspected myocarditis.

Sawalha et al.^6^ found that the mean age of the fourteen COVID‐19 patients was 50.4 years, and the predominant gender was male (58%). Further, in a meta‐summary by Ho et al.,^7^ the mean age of patients with myocarditis was 55 years, and 69% of patients were male. Craver et al.^8^ reported eosinophilic myocarditis in a 17‐year‐old man with COVID‐19 manifestations who died due to cardiac arrest. In the present study, all patients were <60 years old, and complications seemed to be more severe in young patients, and among six patients who were <30 years old, three patients died with age less than 20 years old. Therefore, cardiac monitoring is essential not only in adults but also in youth. However, in the current study, the poor outcome in young patients might also suggest that young age might be a risk factor for more severe cardiac involvement and myocarditis. Although, in contrast to the aforementioned studies, our results showed that the prevalence and severity of disease were higher in females (two of seven female patients died), and further studies with a larger sample size are essential to determine the association between gender and age in COVID 19–induced myocarditis.

Sawalha et al.^6^ noted reduced LVEF in 60% of patients with COVID 19–induced myocarditis. Among fourteen patients reported by Sawalha et al.,^6^ hypokinesia (global or regional; five patients), dilated vena cava (one patient), increased wall thickness (two patients), pericardial effusion (five patients), and tamponade (one patient) were observed. Among nine confirmed myocarditis cases reported by Ho et al.,^7^ six patients had abnormalities on echocardiography, including six cases of reduced LVEF (one severely reduced LVEF), two cases of pericardial effusion, and three cases of left ventricular hypokinesia. Overall, the most common finding was LVEF reduction and LV dysfunction.^6^, ^7^, ^9^ Right ventricle involvement does not appear to be a common manifestation in COVID 19–induced myocarditis.^6^, ^7^ The echocardiographic findings of our patients are consistent with the above findings. Findings included LVEF reduction (eleven patients), hypokinesia (four patients), pericardial effusion (three patients), increased in wall thickness (two patients), and normal RV size and function (nine cases). Increased in PAP (two patients), RV enlargement (three patients), and thrombosis (one patient) were found in lower frequency. Based on our study and other studies, the most common finding was LVEF reduction as well as normal RV size and function. RV involvement (case 2) suggested a probable underlying heart failure that decompensated due to COVID‐19 infection. Furthermore, the current results demonstrated that normal PAP indicated an acute event, leading to a better prognosis. It seemed that increasing LVEF within the first week of treatment could improve the prognosis (Table 2). Moreover, follow‐up of the patients for 3 months exhibited an improvement in LVEF.

**TABLE 2:** Comparing laboratory value at initial presentation and 1 week following treatment shows improvement in left ventricle ejection fraction, NT‐proBNP, and troponin after the first week of treatment

	NT‐proBNP (pg/ml)		LVEF (%)		Troponin (Times UNL)	
	Admission	After 1 week	Admission	After 1 week	Admission	After 1 week
Case 1	21,000	–	10	–	Three	–
Case 2	24,000	3000	5–10	20	Two	Normal
Case 3	35,000	–	20–25	–	Four	–
Case 4	10,000	1500	25	45	Two	Normal
Case 5	24,000	–	15	–	Three	–
Case 6	3000	700	30	40–45	Four	Normal
Case 7	21,000	2400	30–35	40–45	Three	Two
Case 8	4800	600	45	45	Two	Normal
Case 9	12,000	450	30–35	45–50	Two	Normal
Case 10	22,000	800	35	50	Three	Two
Case 11	23,000	600	30–35	45–50	Two	Two

Abbreviations: LVEF, left ventricle ejection fraction; NT‐proBNP, N‐terminal‐pro hormone brain natriuretic peptide; UNL, upper normal limit.

Various studies and case reports suggest different findings on the electrocardiogram, but none of them is pathognomonic. The ECG findings could be without significant changes^10^ or could be associated with non‐specific ST‐segments (diffuse ST‐segment elevation and depression) and T‐wave changes (T‐wave inversion), and ventricular tachycardia.^6^, ^7^ In the cases of our study, the most common ECG findings were ST‐segmental changes (elevation and depression), T‐wave inversion, sinus tachycardia, and low‐voltage waves. Other findings were torsade de points (one patient) and ventricular tachycardia. Thus, it is important to pay attention to serial electrocardiograms. Although ECG findings are not specific, they can give the physicians clues to impending myocarditis in case of fluctuation.

In addition, laboratory findings can be a cost‐effective and simple way to predict severity and outcome in patients with COVID‐19. Gao et al.^11^ noted that the higher levels of N‐terminal prohormone brain natriuretic peptide (NT‐proBNP) might be associated with poor prognosis. Sawalha et al.^6^ reported that troponin was elevated in 91% of cases. Ho et al.^7^ stated that troponin and NT‐proBNP could be elevated or stayed normal (few patients). However, they were elevated in most cases. In the current study, all patients had elevated levels of troponin and NT‐proBNP. High levels of NT‐proBNP and elevated inflammatory markers including IL‐6, CRP, ESR, and WBC were also observed in patients who died. A significant and rapid (within a week) decrease in the levels of cardiac markers was found in the patients who were discharged in a satisfying condition (Table 2). The above results highlight the role of cardiac biomarkers as a good predictive factor for disease severity and outcome. The levels of the markers returned to normal during treatment and follow‐up.

Patients with COVID‐19 are at a higher risk of a hypercoagulable state in both arteries and veins. The thromboembolic disease can be a cause of sudden respiratory failure in these patients. Most patients have high D‐dimer levels indicating sepsis‐induced diffuse intravascular coagulation. Although the role of D‐dimers as a predictive factor for thromboembolic diseases is unknown, elevated D‐dimers have been shown to be an independent risk factor for mortality.^12^ Imaeda et al.^13^ reported a non‐serious COVID‐19 patient with elevated D‐dimers on admission in whom echocardiography and contrast computed tomography revealed a thrombus in dilated left ventricle. Rubartelli et al. reported a COVID‐19 patient with multiple thromboses in the left ventricle, elevated D‐dimers, and severe LVEF reduction of 20% who was followed up for 2 months, and residual thrombosis in the left ventricle together with an LVEF of 16% were observed. In addition, cardiac MRI indicated the findings suggestive of recent myocarditis.^14^ It seems that the ventricular thrombosis that occurs is not a common finding due to COVID‐19. Our case with left ventricle thrombus had elevated D‐dimers and inflammatory markers in addition to CMR suggestive of acute myocarditis. Echocardiography also revealed reduced LVEF (30%–35%). Nevertheless, it is important to think of myocarditis in COVID‐19 patients with ventricle thrombus (especially left ventricle thrombus). Clinical, electrocardiographic, and echocardiographic findings of patients are summarized in Table 3.

**TABLE 3:** Summary of clinical, electrocardiographic, and echocardiographic findings of patients besides their outcome and follow‐up

	Clinical characteristics	Electrocardiogram findings	Echocardiographic findings	Outcome	3 months follow‐up
Case 1	Loss of consciousness, fever, and headache	Sinus tachycardia, low‐voltage QRS	LVEF = 10%, global hypokinesia, normal RV	Died due to cardiac arrest	–
Case 2	Abdominal pain, dyspnea, nausea, and fever	Sinus tachycardia, inverted T wave in lateral limbs and precordial leads	LVEF = 5%–10%, Mild pericardial effusion, RV enlargement,	Survived	LVEF = 20%, dysfunctional and enlarged RV
Case 3	Fever, diarrhea, and dry cough	Normal sinus rhythm, diffuse anterior ST‐segment elevations in precordial leads	LVEF = 20% −25%, Generalized hypokinesia, normal RV size with normal function, dilated inferior vena cava	Died due to cardiac arrest	–
Case 4	Syncope, chest pain, fever, and myalgia	Sinus tachycardia, low‐voltage limb leads, inverted T waves in two limb leads (L1 and aVL), and ST‐segment depression in three pericardial leads	LVEF = 25%, increased wall thickness, normal RV size with mild dysfunction	Survived	LVEF = 45%, normal and functional RV
Case 5	Fever, cough, dyspnea, loss of consciousness	Sinus tachycardia, low voltage limb leads	LVEF = 15%, normal LV and RV size	Died due to ARDS	–
Case 6	Fever, headache	Ventricular tachycardia	LVEF = 30%, normal LV and RV size	Survived	LVEF = 45%–50%
Case 7	Cough, dyspnea	Sinus tachycardia	LVEF = 30%–35%, RV enlargement, left‐sided pleural effusion, dilated inferior vena cava	Survived	LVEF = 45%
Case 8	Dyspnea, pleuritic chest pain	Sinus tachycardia, no significant changes of ST‐segment, low‐voltage waves in limb leads, and QRS alternans	LVEF = 45%, Circumferential pericardial effusion	Survived	LVEF = 45%, mild pericardial effusion
Case 9	Chest pain, fever, cough	Sinus rhythm with ST‐segment elevation and Q waves in precordial leads	LVEF = 30%–35%, Hypokinesia in the anterior wall, normal LV and RV size	Survived	LVEF = 45%–50% and normal cardiac function
Case 10	Fever, cough, cardiopulmonary arrest	Torsade de pointes	LVEF = 35%, normal LV size, increased LV wall thickness	Survived	LVEF = 50%
Case 11	Chest pain, cough, orthopnea	Sinus tachycardia	LVEF = 30%–35%, Mild pericardial effusion, RV enlargement After 3 days: LVEF of 45% and hypermobile clot in the left ventricle	Survived	LVEF = 45%–50%, no signs of embolism

Abbreviations: LV, left ventricle; LVEF, left ventricle ejection fraction; RV, right ventricle.

Given the novelty of this complication and lack of an evidence‐based approach, it appears that the management of COVID 19–induced myocarditis should be similar to the management of other myocarditis etiologies. The American Heart Association (AHA) recommends applying the management algorithm for cardiogenic shock in patients with fulminant myocarditis, including administration of inotropes and/or vasopressors in addition to mechanical ventilation.^15^ Fluid management, prevention of arrhythmias, and inhibition of immune system overactivity are the mainstays of treatment for COVID 19–induced myocarditis.^16^ Heart failure is a complication of myocarditis and may contribute to hemodynamic instability, fluid overload, and pulmonary edema. The use of diuretics, beta blockers, angiotensin‐converting enzyme inhibitors (ACEI) or angiotensin receptor blockers (ARB), and mineralocorticoid receptor antagonists may be clinically indicated.^16^ In addition, the International Society for Heart and Lung Transplantation (ISHLT) recommends the use of advanced therapies for patients when indicated.^17^ Inotropes, intra‐aortic balloon pump, and extracorporeal membrane oxygenation (ECMO) may be required for fulminant myocarditis, cardiogenic shock, and fatal arrhythmias.^18^ Treatment of cardiac arrhythmias could mitigate the adverse effects of myocarditis. Cardiac monitoring leads to early detection of arrhythmias and appropriate treatment, especially bradyarrhythmias and tachyarrhythmias, which are common in COVID 19–induced myocarditis. Bradyarrhythmia may require temporary pacing, and tachyarrhythmia may respond to antiarrhythmic drugs such as lidocaine. Because patients may be prescribed antimalarials and macrolides (QT interval‐prolonging agents), caution should be exercised when taking antiarrhythmics concomitantly.^18^, ^19^

There is also controversy associated with the administration of corticosteroids and IVIG.^18^ A meta‐analysis found that the IVIG can reduce in‐hospital mortality and also significantly improve LVEF. Furthermore, the efficacy of IVIG administration was greater in acute fulminant myocarditis.^20^ Li et al.^21^ also found that IVIG reduced mortality and improved LVEF in children with myocarditis. However, corticosteroids indicated no beneficial effect in their study. The rapid improvement in cardiac function and structure without a significant decrease in viral load may suggest that the cytokine storm (immune system) plays a role in COVID‐19 myocarditis that is independent of viral replication.^22^ Although there are no established guidelines for treatment of COVID 19–induced myocarditis, the present study considered the administration of corticosteroids and IVIG because of the immunologic nature of myocarditis. We administered high‐dose corticosteroid (1 g/day) to six patients with critical situations such as cardiogenic shock, low blood pressure, or life‐threatening arrhythmias, of whom three were discharged and three died. Besides, three of these patients received IVIG (one of them had an LVEF of 10%, and two of them had life‐threatening arrhythmias) and one of them was discharged and two of them died. More studies are necessary to prove the beneficial effects of high‐dose corticosteroids and IVIG. In the absence of well‐established guideline, the ongoing study recommends administration of high‐dose corticosteroids as well as IVIG in severe critically ill patients, who do not respond to usual treatment modalities.

The present study had one important limitation. None of the patients had an endomyocardial biopsy, which is the standard method for diagnosing myocarditis, and only one patient had cardiac MRI. Therefore, the diagnosis of myocarditis was not definite in any of them. However, the clinical and laboratory findings supported a diagnosis of myocarditis.

## 14 CONCLUSION

Myocarditis is a life‐threatening complication of COVID‐19 that can worsen the patient's condition and lead to morbidity and mortality. The current study presents eleven cases who were clinically suspicious of COVID 19–induced myocarditis along with their laboratory and imaging findings. In our cases, females and younger patients developed more severe disease. In contrast, improvement in LVEF and proBNP within the first week of treatment contributed to promising outcomes. However, more studies are necessary to define the role of age and gender in the prognosis of COVID 19–related myocarditis. Therefore, a deep understanding of the possible manifestations of COVID 19–induced myocarditis leads to early diagnosis and appropriate management of the disease. Although it seems that COVID‐19 myocarditis is not a common complication, its lethal nature makes it essential to carefully monitor patients for possible clinical and laboratory findings of myocarditis (especially females and younger ages). Further studies are needed to determine the prevalence, underlying mechanism, and proper management of this potentially lethal condition.

## CONFLICT OF INTEREST

The authors declare no conflict of interest.

## AUTHOR CONTRIBUTIONS

Ahmad Amin conceptualized the study. Seyed Parsa Eftekhar prepared the first draft of the manuscript. Ahmad Amin and Seyed Parsa Eftekhar revised and edited the first draft of the manuscript. Naghmeh Ziaie critically revised the manuscript and supervised the project. All authors participated in the preparation of the data. All authors read the final draft of the manuscript and approved it.

## ETHICAL APPROVAL

This study was approved by the Ethics Committee of Babol University of Medical Sciences (Babol, Iran).

## CONSENT

Written informed consent was obtained from the patients to publish this report in accordance with the journal's patient consent policy.

## Data Availability

The data supporting the findings of this study are available on request from the corresponding author and with permission from Babol University of Medical Sciences, Babol, Iran.
